# Evaluation of the Effect of Low-dose Aspirin on the Prevention of Preterm Delivery in Women with a History of Spontaneous Preterm Delivery

**DOI:** 10.1055/s-0043-1772480

**Published:** 2023-11-29

**Authors:** Masoumeh Mirzamoradi, Zahra Dehghani, Pegah Azadi, Maryam Mohammadi, Armin Khavandegar, Mahmood Bakhtiyari

**Affiliations:** 1 Department of Obstetrics and Gynecology, Shahid Beheshti University of Medical Sciences, Tehran, Iran; 2 Sina Trauma and Surgery Research Center, Sina Hospital, Tehran University of Medical Sciences, Tehran, Iran; 3 Non-communicable Diseases Research Center, Alborz University of Medical Sciences, Karaj, Iran

**Keywords:** spontaneous PTL, PTD, preterm premature rupture of the membrane, low-dose aspirin, PTL espontâneo, PTD, ruptura prematura da membrana, aspirina em baixa dosagem

## Abstract

**Objective** Currently, uteroplacental vascular disorders are considered one of the main mechanisms of spontaneous preterm delivery (PTD). Low-dose aspirin is used to prevent pre-eclampsia, which has a similar mechanism; hence, the present study aimed to investigate the effect of low-dose aspirin on the prevention of PTD in women with a history of spontaneous PTD.

**Methods** The present pilot randomized clinical trial was conducted on 54 pregnant women in the aspirin group (taking 80 mg daily until the 36 ^th^ week and classic treatment) and 53 patients in the control group (only receiving classic treatment).

**Results** Forty-three patients (40%) presented before 37 weeks due to symptoms of PTL. Preterm delivery (< 37 weeks) occurred in 28 patients (26%), and there was no significant difference between the aspirin and control groups (10 patients [19%] and 18 patients [34%], respectively; *p* = 0.069). The time of preterm delivery was early (< 34 weeks) in 6 patients (21%), and its cause was spontaneous labor in 23 patients (82%) which was not significantly different between the two groups ( *p* > 0.05). Out of 40 patients with spontaneous labor, 25 patients (63%) had a PTD, which was significantly lower in the aspirin group than in the control group (9 patients [45%] versus 16 patients [80%], respectively; *p* = 0.022).

**Conclusion** The findings of the present study demonstrated that despite the reduction in the incidence of PTD using low-dose aspirin, the reduction rate was not statistically significant. On the other hand, in patients with spontaneous labor prone to PTD, aspirin was effective in reducing the incidence of PTD.

## Resumo

**Objetivo** Atualmente, os distúrbios vasculares uteroplacentários são considerados um dos principais mecanismos de parto prematuro espontâneo (PTD). A aspirina em baixa dose é usada para prevenir a pré-eclâmpsia, que tem um mecanismo semelhante; portanto, o presente estudo teve como objetivo investigar o efeito da aspirina em baixa dosagem na prevenção de PTD em mulheres com história de PTD espontâneo.

**Métodos** O presente ensaio clínico piloto randomizado foi realizado em 54 gestantes do grupo aspirina (tomando 80 mg diários até a 36ª semana e tratamento clássico) e 53 pacientes do grupo controle (somente tratamento clássico).

**Resultados** Quarenta e três pacientes (40%) apresentaram-se antes de 37 semanas devido a sintomas de PTL. O parto prematuro (< 37 semanas) ocorreu em 28 pacientes (26%) e não houve diferença significativa entre os grupos aspirina e controle (10 pacientes [19%] e 18 pacientes [34%], respectivamente; p = 0,069). O tempo de parto prematuro foi precoce (< 34 semanas) em 6 pacientes (21%) e sua causa foi trabalho de parto espontâneo em 23 pacientes (82%) que não foi significativamente diferente entre os dois grupos (p > 0,05). Das 40 pacientes com trabalho de parto espontâneo, 25 pacientes (63%) tiveram PTD, que foi significativamente menor no grupo aspirina do que no grupo controle (9 pacientes [45%] versus 16 pacientes [80%], respectivamente; p = 0,022).

**Conclusão** Os achados do presente estudo demonstraram que, apesar da redução na incidência de DPT com o uso de aspirina em baixa dosagem, a taxa de redução não foi estatisticamente significativa. Por outro lado, em pacientes com trabalho de parto espontâneo propensas a PTD, a aspirina foi eficaz na redução da incidência de PTD.

## Introduction

Preterm labor (PTL) before the 37 ^th^ week of gestation is the most common cause of worldwide morbidity and mortality in newborns. 1 The prevalence of PTL in developed and developing countries is 5 and 25%, respectively. 2 Preterm labor is responsible for 75% of all cases of neonatal mortality and for 40% of all cases of neurologic neonatal morbidities. 3 Since currently there is no effective medical treatment for termination of the PTL process, the best and most reasonable solution is to identify women at risk and utilize preventive medical interventions. 4

Despite an intensive bulk of studies on PTL pathophysiology, there is still much controversy on its mechanism. One of the most acceptable theories is bypassing or early stimulation of “parturition complex cascade”, known to be responsible for on-time labor triggering. 5 6 7 Besides, labor is believed to be a proinflammatory event. Preterm labor as an overwhelming inflammatory event occurring earlier in the midtrimester has been reported to be followed by an intraamniotic infection in almost 50% of PTL cases. 8 9

The most important risk factors for preterm delivery (PTD) are a previous history of PTD, smoking, vaginal bleeding, and preterm premature rupture of the membrane (PPROM). 4 The most important risk factor is a history of previous PTL. 10 There is a bulk of studies that report that placental uterine ischemia plays a crucial role in spontaneous PTL 11 12 13 14 and women with a PTL history are at a higher risk of cardiovascular disease in the future. 15 16 These findings indicate that the mechanism of PTL is similar to that of other ischemic placental diseases such as pre-eclampsia. Low-dose aspirin is used to inhibit platelet aggregation and prevent pre-eclampsia. 17

Due to the similarity between the mechanisms of spontaneous PTL and pre-eclampsia, it has been suggested that low-dose aspirin may also be used to prevent spontaneous PTL. Some studies have performed secondary analyses of data on the effect of low-dose aspirin on the prevention of pre-eclampsia to evaluate the effect of low-dose aspirin on the prevention of PTL. Although some studies have reported the effectiveness of low-dose aspirin in preventing PTL, other studies did not prove it. 18 19 20 21 Moreover, a randomized clinical trial was conducted to compare the effect of low-dose aspirin on the incidence of PTL, 2 but its clinical results have not been reported yet. Therefore, it seems that no independent study has been conducted in this field so far, and the results of studies on the effect of low-dose aspirin on the prevention of PTL are secondary analyses in patients with risk factors for pre-eclampsia. Therefore, the present study aimed at evaluating the effect of aspirin on the prevention of PTD in women with a history of preterm delivery.

## Methods

The present randomized clinical trial was conducted as a pilot study on pregnant women with a history of PTD who were referred to the Mahdieh and Shohada Tajrish hospitals in Tehran, Iran, in 2019 and 2020. The inclusion criteria consisted of age > 18 years old, singleton pregnancy, gestational age of 8 to 16 weeks, and a history of spontaneous PTD. The exclusion criteria were the following: twin or multiple pregnancies, history of non-spontaneous PTD due to maternal problems such as pre-eclampsia, HELLP syndrome, or fetal problems such as intrauterine growth restriction (IUGR), taking aspirin for treating other diseases, including vasculitis, lupus, type 1 and 2 diabetes, hypertension kidney problems, and antiphospholipid syndrome. Thrombocytopenia, fetal malformations in the current pregnancy or previous PTD, and simultaneous participation in another trial were other exclusion criteria.

Using the following formula and considering the likely incidence of PTD of 15%, α = 0.05, and d = 0.1 (10% accuracy), the calculated sample size was 50 people in each group



**Fig. 1:**
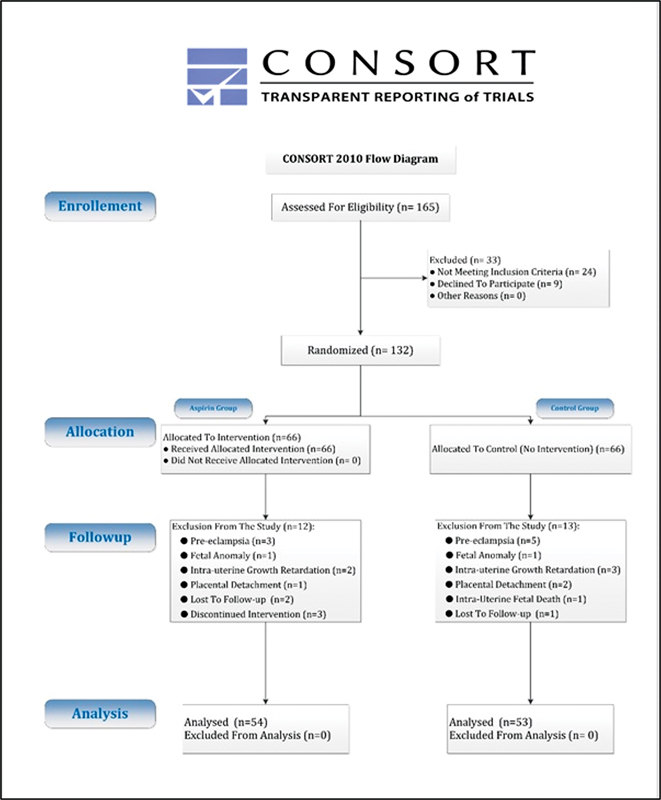
Consort algorithm of our study.

Considering a patient dropout of 30%, the required sample size for each group increased to 66 patients in each group. Eligible patients were selected via convenience sampling in the order of referral, and after explaining the study method and obtaining written consent, the subjects were enrolled in the study. Utilizing a random number table, the patients were divided into two groups: the intervention group, taking aspirin with a dosage of 80 mg daily, and the control group, without intervention. During the study, 12 patients in the aspirin group and 13 patients in the control group withdrew, and eventually, 54 patients in the aspirin group and 53 patients in the control group completed the study, and their data were analyzed ( Figure 1 ).

Using a form, we collected the data on patients, including age, history of pregnancy (gravidity, parity, history of abortion and stillbirth, number of alive and dead children), etiology for previous spontaneous PTD (spontaneous labor, PPROM), and PTD category based on the fetal age (early: < 34 weeks, late: from 34 to 37 weeks). Then, the patients were randomly divided into two groups. Patients in the aspirin group took an 80 mg tablet, manufactured by Hakim Pharmaceutical Company, Iran, daily and preferably in the evening until the 36 ^th^ week of pregnancy, in addition to the classic treatment. The control group received only classic treatment for PTL. All patients received the classic treatment, including receiving preventative weekly injections of Proluton (from the 16 ^th^ to the 36 ^th^ week). 17-alpha-hydroxyprogesterone caproate 250 mg, also known as Proluton 250 mg, is administered intramuscularly on a weekly basis to inhibit PTL contractions. Routine pregnancy visits were performed for both groups. Moreover, in cases with a cervix length < 25 mm, cervical cerclage or pessary was used. In the presence of PTL symptoms. such as spontaneous labor or PPROM, tocolytic (at 33 weeks or less) 22 and betamethasone (at 37 weeks or less) 23 24 were used if indicated according to available studies. However, at 34 to 37 weeks of gestation, betamethasone was not used if there was an impending delivery or for diabetic mothers. Gestational age at delivery and characteristics of the infant (sex and Apgar scores at minutes 1 and 5), presence of symptoms of PTL, administration of betamethasone, administration of tocolytic agents and its type, PTD cause, infant need for neonatal intensive care unit (NICU) and duration of stay in NICU were also recorded. Finally, the incidence of PTD (in < 37 weeks) and its time and causes were compared between the two groups. IBM SPSS Statistics for Windows version 25 (IBM Corp., Armonk, NY, USA) was used for data entry and analysis. Qualitative variables were described using frequency and percentage, and quantitative variables were described using the mean and standard deviation (SD) or median and interquartile range (IQR). The chi-squared test, the Fisher Exact test, the independent t-test, and the Mann-Whitney U test were used to analyze the data. The significance level was set as *p* < 0.05.

All methods used in the present study are based on the principles of Helsinki Human Studies. Patient information will be kept entirely confidential during and after the study. The study was approved by the ethics committee of Shahid Beheshti University of Medical Sciences (Registration code: IR.SBMU.MSP.REC.1398.528) and was registered in the site of the Iranian registry of clinical trials ( www.irct.ir ) (Registration code: IRCT20191031045289N1).

## Results

Table 1 compares the two groups, aspirin and control, in terms of the characteristics of the patients. Pessary and cervical cerclage, respectively, were used in 4 patients (4%) and 16 patients (15%), and there was no significant difference between the two groups ( *p* > 0.05). Only two stillbirths occurred in the present study, one in the case group and one in the control group. Forty-three patients (40%) presented symptoms of PTL before 37 weeks, which was not significantly different between the aspirin and control groups (21 patients [39%] and 22 patients [42%], respectively; *p* = 0.782). Among them, the cause of referral was spontaneous labor in 40 patients (93%) (20 patients in each group) and PPROM in 3 patients (7%) (1 patient in the aspirin group and 2 patients in the control group). Among all patients, a betamethasone injection was applied in 40 patients (37%), and there was no significant difference between the aspirin group and control group (18 patients [33%] versus 22 patients [42%]; *p* = 0.382). In three patients, betamethasone was not used due to impending delivery or maternal diabetes. In general, tocolytic agents were used in 16 (40%) patients, that is: magnesium sulfate in 8 patients, indomethacin in 7 patients, and nifedipine in 1 patient. There was no significant difference between the aspirin and control groups in terms of the frequency of tocolytic administration (6 patients [11%] versus 10 patients [19%], respectively; *p* = 0.261). Preterm delivery occurred in 28 patients (26%), and there was no significant difference between the aspirin group and the control group (10 patients [19%] versus 18 patients [34%]; *p* = 0.069). The time of PTD was early in 6 patients (21%), and its cause was spontaneous labor in 23 patients (82%), which was not significantly different between the aspirin group and control group ( Table 2 ) ( *p* > 0.05). Out of 40 patients with spontaneous labor, despite using methods for preventing PTL, 25 patients (63%) had PTD, which was significantly lower in the aspirin group than in the control group ( *p* = 0.022) ( Table 3 ).

**Table 1:** Comparison of patient characteristics between the two groups

Variables		Aspirin group ( *n* = 54)	Control group ( *n* = 53)	*p-value*
Age (years old)	Mean ± SD	31 ± 5	30 ± 5	0.134 **†**
Gravity	2	21 (39%)	23 (44%)	0.891‡
	3-4	25 (46%)	23 (44%)	
	≥5	8 (15%)	7 (12%)	
Abortion history	None	34 (63%)	34 (64%)	0.701‡
	1	9 (17%)	12 (23%)	
	2	7 (13%)	5 (9%)	
	≥3	4 (7%)	2 (4%)	
Children count	None	9 (17%)	11 (21%)	0.667‡
	1	31 (57%)	30 (56%)	
	≥2	14 (26%)	12 (23%)	
Dead child history	No	37 (68%)	37 (70%)	0.609‡
	Yes	17 (32%)	16 (30%)	
Previous PTD cause	Spontaneous labor	48 (89%)	42 (79%)	0.172‡
	PPROM	6 (11%)	11 (21%)	
Time of previous PTD	Early (< 34 weeks)	31 (57%)	28 (53%)	0.634‡
	Late (34–37 weeks)	23 (43%)	25 (47%)	

Abbreviations: PTD: preterm delivery; PPROM: preterm premature rupture of membrane; SD: standard deviation.

† Independent t test

‡ Chi-squared test;

**Table 2:** Comparison of preterm delivery characteristics between the two groups

	Aspirin group ( *n* = 10)	Control group ( *n* = 18)		*p-value* †
PTD based on fetal age	Early (< 34 weeks)	2 (20%)	4 (22%)	1.000
	Late (34–37 weeks)	8 (80%)	14 (78%)	
PTD etiology	Spontaneous labor	7 (70%)	16 (89%)	0.315
	PPROM	3 (30%)	2 (11%)	

Abbreviations: PTD: preterm delivery; PPROM: preterm premature rupture of membrane.

† Fisher Exact test;

**Table 3:** Comparison of preterm delivery characteristics in patients referred due to spontaneous labor between the two groups

	Aspirin group ( *n* = 20)	Control group ( *n* = 20)	*p-value*
Betamethasone administration	17 (85%)	20 (100%)	0.231*
Tocolytic agents administration	6 (30%)	8 (40%)	0.507**
PTD	9 (45%)	16 (80%)	0.022**

Abbreviation: PTD: Preterm delivery.

*Fisher Exact test

**Chi-squared test

In 16 patients receiving tocolytic agents, the IQR and median duration of prolongation of pregnancy were 1 to 7 weeks and 3.5 weeks, respectively. The effect of tocolytic factors on the prolongation of pregnancy in the aspirin group was significantly higher than that in the control group (7 weeks versus 2 weeks, respectively; *p* = 0.007). Table 4 also presents the characteristics of infants.

**Table 4:** Comparison of neonatal characteristics between the two groups

		Aspirin group ( *n* = 54)	Control group ( *n* = 53)	*p-value*
Newborn sex	Girl	30 (56%)	35 (66%)	0.276†
	Boy	24 (44%)	18 (34%)	
Apgar 1 ^st^ minute	≤6	0 (0%)	1 (2%)	0.482‡
	7–8	6 (11%)	8 (15%)	
	9	48 (89%)	44 (83%)	
Apgar 5 ^th^ minute	≤8	0 (0%)	1 (2%)	0.641‡
	9	5 (9%)	6 (11%)	
	10	49 (91%)	46 (87%)	
NICU admission		10 (19%)	17 (32%)	0.106‡
		Aspirin group ( *n* = 10)	Control group ( *n* = 17)	***p-value***
NICU admission cause	Prematurity, RDS or TTN	7 (70%)	16 (94%)	0.128‡
	Others	3 (30%)	1 (6%)	
NICU stay	1–2 days	4 (40%)	9 (53%)	0.849‡
	> 3 days	6 (60%)	8 (47%)	

Abbreviations: NICU, neonatal intensive care unit; RDS: respiratory distress syndrome; TTN: transient neonatal. tachypnea

† Fisher Exact test

‡ Chi-squared test

## Discussion

In the present study, we aimed to assess the preventive effect of low-dose aspirin on PTL in pregnant women with a history of PTD. Although not being statistically significant, the findings of the present pilot study revealed that low-dose aspirin prescription in women with a history of PTD reduced the incidence of PTD. Moreover, we realized that in patients with a history of spontaneous PTD, receiving low-dose aspirin significantly reduced PTD if the patient experienced spontaneous labor in the current pregnancy.

In a study by Hoffman et al. on pregnant women from low- to middle-income countries, low-dose aspiring showed reduced PTD prior to 37 weeks, in addition to decreased perinatal mortality. 25 In contrast, in a study published in 2022 encompassing 608 pregnant women with a history of spontaneous preterm birth, low-dose aspiring did not demonstrate a significant reduction in preterm birth. 26

In studies investigating low-dose aspirin to prevent pre-eclampsia, a decrease in PTL is also reported. Nevertheless, the cause of PTL has not been reported generally, and apparently, a decrease in PTL could be due to the decrease in the incidence of pre-eclampsia as one of the important causes of PTL. 27 However, part of the reduction in the incidence of PTD reported in these studies may be attributed to aspirin use. On the other hand, currently published studies on the role of aspirin in the prevention of PTD are mainly secondary analyses of studies and clinical trials on aspirin use for controlling pre-eclampsia, some of which reported beneficial effects of aspirin in the prevention of PTD; other studies did not prove that. Allshouse et al. conducted a reanalysis of data obtained from a clinical trial that investigated the effects of aspirin on women at high risk for pre-eclampsia. 19 They reported that high-risk women with pre-eclampsia who had been receiving aspirin had a reduced rate of PTL due to spontaneous labor or PPROM compared with a placebo group; however, the difference was not statistically significant. Van Vliet et al. conducted a meta-analysis of the data of participants in studies investigating the effect of antiplatelet agents on reducing pre-eclampsia and reported that women who took antiplatelet agents, as compared with a placebo or no treatment group, had a lower risk for PTL in < 37 and 34 weeks of gestation. 20 Although their results indicated the beneficial effects of antiplatelet agents on reducing spontaneous PTL in women at risk for preeclampsia, it may not be effective in cases where such risk factors are not present. Visser et al. also conducted a multicenter randomized clinical trial to determine the effect of low-dose aspirin on preventing spontaneous PTL in women with a history of PTL. 2 Nevertheless, it seems that the clinical results of the mentioned study have not been published yet. Finally, Andrikopoulou et al. conducted a secondary analysis of data obtained from a randomized clinical trial on the effect of low-dose aspirin on the prevention of pre-eclampsia in low-risk nulliparous women and reported a significant reduction in spontaneous PTL in < 34 weeks. However, the incidence of PTL from the 34 ^th^ to the 37 ^th^ week and spontaneous PTL in < 37 weeks were not statistically significant. 21

The results of the aforementioned studies suggest that, in some cases, low-dose aspirin may be effective in preventing spontaneous PTL, although those studies were secondary analyzes in women with risk factors for pre-eclampsia. Although our study, an independent study on women at risk for PTL, was associated with a reduced incidence of PTD in the aspirin group, there was no statistically significant difference. On the other hand, the median duration of prolongation of pregnancy was significantly higher in patients receiving tocolytic agents in the aspirin group. However, because the incidence of PTD was significantly lower in the aspirin group in a subgroup of patients who presented with spontaneous labor in the current pregnancy and were at risk for PTD, it may not be significant in all patients due to the small sample size.

No independent study has been conducted to investigate the effect of low-dose aspirin on the prevention of spontaneous PTL, and studies in this field have been the secondary analyses of research conducted on women with risk factors for pre-eclampsia. The latest recommendations of the American Association of Obstetricians and Gynecologists also indicate that currently, due to lack of sufficient evidence, prophylactic administration of low-dose aspirin has not been approved for the prevention of conditions such as PTL and stillbirth, among others, in the absence of risk factors for pre-eclampsia. 28 Hence, as the strength of our study, it was conducted as an independent randomized clinical trial on the effect of low-dose aspirin for controlling PTD due to spontaneous labor or PPROM. However, our results showed that low-dose aspirin is not effective in preventing spontaneous PTD in women with a previous history of PTD caused due to spontaneous labor or PPROM.

However, the small sample size, which was the most significant limitation of our study, may have provided statistically insignificant results. In addition, due to not using a placebo in our research, there was no blinding. Therefore, it is recommended to conduct extensive multicenter studies with larger sample sizes and utilize a placebo.

Despite our effort to provide a rationale for using low-dose aspirin in PTL, there were some limitations to the present study. The main limiting factor in the present study is the small sample size, eclipsing its representativeness. Further studies are needed to evaluate the effectiveness of low-dose aspirin in larger populations. Besides, as we were concerned about the term “proven therapy” as defined in the Helsinki declaration, we refused to expose patients in the control group to placebo drugs. 29 Besides, since pregnancy is a very delicate and sensitive issue, most patients would have refused to use a “a not-knowing-what-it-is drug”. Recently, there have been some concerns regarding the placebo usage even in the normal population. 29 30 Further studies are needed to assess the placebo effect in a larger population of PTL cases.

## Conclusion

The findings of the present study showed that despite the reduction in the incidence of PTD using low-dose aspirin, the reduction rate was not statistically significant. On the other hand, in patients with spontaneous labor prone to PTD, aspirin was effective in reducing the incidence of PTD. Therefore, conducting more extensive studies with larger sample sizes may produce significant results.
